# Does intellectual capital drive bank's performance in Bangladesh? *Evidence from static and dynamic approach*

**DOI:** 10.1016/j.heliyon.2023.e17656

**Published:** 2023-06-27

**Authors:** Mohammad Omar Faruq, Tamanna Akter, Mohammed Mizanur Rahman

**Affiliations:** a Department of Accounting and Information Systems, Faculty of Business Studies, Comilla University, Cumilla 3506, Bangladesh; b Department of Accounting and Information Systems, Faculty of Business Studies, Jahangirnagar University, Savar, Dhaka 1342, Bangladesh

**Keywords:** MVAIC, HCE, SCE, RCE, CEE, Firm performance, Panel data, Two-step system GMM, Bangladesh

## Abstract

This paper investigates the relationship between measures of intellectual capital efficiency and the performance of the listed banks in Bangladesh. We have collected data from the listed bank's published annual reports for seven years (2015–2021). We have primarily used standard panel data analysis techniques to assess the static relationships. In addition, to the static models, we have also checked the dynamic models for robustness in the context of Bangladesh. Following the RBV (Resource Based theory), we have found that the MVAIC (Modified Value-Added Intellectual Coefficient) significantly and positively determines firm performance in both static and dynamic methods. However, various components of MVAIC show differing relationships, which indicates that the two-step system GMM (Generalized Method of Moments) is superior to static models. The moderating role of MEETING (Company Meetings) is significant with respect to MVAIC. In addition, the moderating role of corporate governance variables at the component level remained the same in both methods. Our further analysis of the association with respect to the before and during pandemic periods suggests that the relationship remains the same irrespective of the period under study. Future research can use this paper to understand the significance of dynamic modeling while studying IC (Intellectual Capital) in an emerging economy context. This is among a few studies that have applied both static and dynamic models to assess the relationship between IC and firm performance in the context of emerging economies. Policymakers and bank managers in Bangladesh could use the findings of this study to realize that IC is much more valuable than other available tangible assets in creating a sustainable competitive advantage.

## 1 Introduction

Global competition, along with the development of science and technology, has enabled the rapid growth of international business [1]. Today's world is knowledge-based and technology-driven [2]. IC (Intellectual Capital) is a predictor of firm performance and a source of competitive advantage [3]. Service industries contribute dominantly to economic development [4]. IC transforms an economy from an industrial one to a knowledge-intensive one [4]. Reference [5] described the inability of firms to properly manage due to the difficulties in measuring IC. Intellectual capital is a valuable source of firm performance and competitive advantages when measured accurately. There has been a plethora of research assessing the relationship between the VAIC (Value Added Intellectual Coefficients) and various measures of firm performance [[6], [7], [8], [9]]. In addition, most of the studies have assessed the relationship with several accounting-based measures of performance [1,3,10]. However, the availability of fine-tuned research in Asia on the link between IC efficiency and firm performance is not worth mentioning [11]. Furthermore, the authors mentioned that the inclusion of the COVID (Corona Virus Disease) pandemic is also incomplete. Emerging economies like Bangladesh are an excellent sample for studying the relationship between performance and VAIC. In addition, relationships between the VAIC and banking industries will provide information regarding the status of value creation within the banking industry.

Research on IC in Bangladesh is still in its infancy, but it has a lot to offer. Thus, we selected Bangladesh as an ideal laboratory to investigate our research question. After reviewing various recent works on the relationship between IC and firm performance, we have found several research gaps to address. First of all, most of the studies use the controversial VAIC model while representing IC [[12], [13], [14], [15], [16], [17]]. Then, the use of corporate governance variables as controls has long been ignored and incomplete. Although the moderating role of corporate governance variables has been studied in other economies, there has been no mentionable research in Bangladesh. One final gap is that the study models used in Bangladesh in the past were mostly static, whereas the use of dynamic models reveals more accurate and unbiased results. The relationship will be assessed by answering the following questions: Does intellectual capital efficiency affect the listed banks performance in Bangladesh using both static and dynamic parameter estimation models? Do intellectual capital efficiency components affect the listed banks performance in Bangladesh using both static and dynamic parameter estimation models?

The rationale behind this study is that it contributes to the existing literature and addresses several research gaps with the available dataset. First, MVAIC is used to measure the IC efficiency, which is more accurate and consistent than the traditional VAIC model and overcomes the limitations of past studies conducted in Bangladesh. Second, this study uses various corporate governance attributes to control for the presence of any significant influence from the differing governance structures of the listed banks in Bangladesh. Third, this study further examines the role of corporate governance variables as moderators of the relationship between IC and performance. Finally, this paper shows concern over the widely used static models in Bangladesh and tests their validity through the use of a dynamic and more robust parameter estimation model.

RBV (Resource Based theory) provided by Ref. [18] has been extensively referred to in the literature while studying the relationship between IC and firm performance. Further references to the RBV were directed toward the reliance on intangible assets [19]. We have also used RBV as our baseline theory for justifying the relationship under study. Following the limitations and suggestions provided by authors who have tested the relationship between IC and firm performance, we have developed our hypothesis along with several corporate governance controls for clarity [20,17]. In addition, we have used a more recent and longer period of data for our study, including the advent of COVID-19 (Corona Virus Disease-2019). We have used panel data analysis using FE (Fixed Effect) and RE (Random Effect) analyses. Finally, we have also assessed the dynamic two-step system GMM with robust standard errors to cross-check the validity of our static models.

The remainder of the study is organized as follows: Section 2 reviews the past published literature and assumes several hypotheses based on the past studies. The data and methodology used in the study are discussed in the next section. Then, under Section 4, we have organized our results and discussions. In the final section, we have concluded our investigation regarding future research recommendations and a few of the limitations present in our study.

## 2 Literature review and hypotheses development

### 2.1 Primer on intellectual capital

[21] first coined the term IC (Intellectual Capital) as the value creation process. Several other authors have tried to define the term IC [22,23]. Reference [24] defined IC as a vital resource for firms in creating value. IC comprises three aspects of intangibles: human competence and internal and external structures [25]. Reference [26] sees IC as knowledge to be transformed into value. On the other hand, Ref. [27] defined IC as an investment in the form of human capital. Reference [28] defines IC as the combination of human, information, and organizational capital. Nevertheless, all the authors agree on one thing: intangible assets are the source of wealth generation for firms. Most IC definitions are directed toward categorizing the broader definition into several dimensions for clarity [25,[29], [30], [31]]. Reference [32] noted IC as “unaccounted capital” in the traditional accounting system. IC is frequently defined vaguely [33]. However, the definitions of IC originate from different disciplines, which creates a lack of convergence. Reference [34] suggested that the need for further development and classification of the IC framework should be ignored and that practitioners and academicians should look for convergence. The field of studying IC is still dynamic and capable of bringing a new debate or contribution to the previous findings [35].

### 2.2 Theories relating to firm performance and IC

#### 2.2.1 RBV (Resource Based theory)

The resource-based theory is widely used in explaining the relationship between firm performance and intellectual capital [36]. Firm resources are considered the key driver of firm performance [18]. These resources include both physical and intangible assets that firms use to stay competitive and profitable [37]. Reference [18] places specific importance on the uniqueness of the firm's resources to achieve competitive advantages. Explicitly, the uniqueness of intangible assets ensures long term competitiveness. However, it is also important to note that both physical and intangible assets depend on each other in terms of performance. The theory suggests that internal resources can generate value for firms if used effectively [36]. In the past, the strategic resources of a firm were referred to using only tangible and financial assets. But in recent times, in a knowledge-driven economy, the focus has been more on the intangibles. IC improves the chance of a sustainable competitive advantage as it cannot be easily replicated by competitors, which can be done for other physical assets [36,38]. Reference [39] also supports the argument and suggests that IC drives value creation in this era of knowledge. In another note, the role of IC is not limited to certain distinctions: small and large firms or developed and developing countries. Following this argument, we have the motivation to test the relationship in Bangladesh. Studies conducted in different parts of the world have shown the positive influence of IC on firm performance.

#### 2.2.2 Resource dependency theory

Ownership of external resources ensures the survival of any firm, which is quite uncertain and poses a threat. Without continued access to external resources, firms will lose their strategic importance. Furthermore, the possibility of owning all the external resources is quite impractical [40]. However, a link between the external factors and the corporate boards can mitigate such a situation. The central assumption of the resource dependence theory is that corporate governance structure and composition of the board can be considered vital resource [41]. External resources that are essential for corporate success can be explored easily through the corporate boards. Several studies have studied the impact of corporate governance on the performance of a firm and found a positive relationship.

### 2.3 Financial performance and IC in the banking industry

Emerging economies depend vastly on banks for loans [42]. The growth of national markets is also based on the financial transactions facilitated by banks in emerging economies [43]. The bank's competitive advantages greatly depend on intangibles over physical and financial assets in the contemporary knowledge-intensive economy [44]. Past studies have categorized the banking industry as knowledge-based and suggested IC as a key predictor of bank profitability [45]. In addition, Ref. [46] emphasized the need for intellectual and physical assets. However, banks are highly competitive service sectors, for which they face difficulties in supervising their intellectual assets [44]. Several authors have examined the relationship between VAIC and bank performance with mixed results in different country settings [4,[6], [7], [8], [9],^17^,^42^,^43^,[47], [48], [49], [50], [51], [52]]. Multiple studies in the past have observed a significant direct relationship between IC and firm performance but mixed results at the component level [43]. Re-examining the relationship between the IC and a firm's performance in country-specific settings can be of greater value [7]. From Table 1 it is evident that several studies were undertaken in Bangladesh to assess the relationship between IC and firm performance [20,53]. More specifically [17] also studied Bangladesh's banking sector using the available data for the listed banks from 2014 to 2018. However, all these studies investigate the role of IC on firm performance without considering the dynamic aspects, which may produce biased results. Various studies have claimed that the dynamic aspects of the relationships should also be checked along with the static models, as the impact of the past year's value may have a significant impact on the current value of the dependent variables [54,55]. In addition, they have only used samples covering five years before the advent of the COVID-19 pandemic, for which there is a need for re-examination. On another note, most of the studies performed under the Bangladesh perspective ignore the impact of other corporate governance variables, which are essential to allow for market differences [56] noted differences between developed and emerging countries due to the varying nature of the markets. We have used corporate governance controls to allow for the differences present among firms and country-specific regulatory requirements.

**Table 1:** Overview of the previously published literature (IC and Firm performance).

Authors	Sample	Time period	Econometric Methods applied	IC Valuation	Measure of performance
Bayraktaroglu et al. (2019)	Manufacturing firms (Turkey)	2003–2013	Multiple Regression	VAIC	ROA, ATO, ROE, MB
Ginesti et al. (2018)	Non-listed companies (Italy)	2016	OLS regression	VAIC	ROA, ROE, ROI, ATO
Nirino et al. (2022)	European firms	2017–2018	OLS regression	VAIC	Tobin's Q
Oppong and Pattanayak (2019)	Commercial banks (India)	2006–2017	Panel Data (Fixed and Random effect)	VAIC	EP, ATO
Singla (2020).	Real Estate and infrastructure firms (India)	2008–2017	Panel Data (Fixed and Random effect)	VAIC	ROA, PB
Smriti and Das (2018)	Listed companies (India)	2001–2016	System GMM	VAIC	ATO, ROA, Tobin's Q, SG
Xu and Li (2019)	SMEs (China)	2012–2016	Multiple Regression	MVAIC	EBIT, ROA, NPM, ATO
Xu and Li (2022)	Manufacturing sector (China)	2012–2016	Multiple Regression	MVAIC	EBIT, ROA, ROE, ATO
Ting et al. (2020)	Electronic companies (Taiwan)	2006–2017	OLS regression	VAIC	Efficiency, Change in Sales
Chowdhury et al. (2019)	Pharmaceutical companies (Bangladesh)	2013–2017	Multiple Regression	VAIC	ATO, ROA, ROE, MB
Soewarno and Tjahjadi (2020)	Banking firms (Indonesia)	2012–2017	Multiple Regression	VAIC, A-VAIC	ROA, ROE, ATO, PBV
Mollah and Rouf (2022)	Commercial banks (Bangladesh)	2014–2018	Multiple Regression	VAIC	ROA, ROE, RG
Clarke et al. (2011)	Listed companies (Australia)	2004–2008	OLS regression	VAIC	ROA, ROE, RG, EP
Nadeem et al. (2017)	BRICS economies	2005–2014	System GMM	VAIC	ROA, ROE, ATO, P/B
Phusavat et al. (2011)	Manufacturing firms (Thailand)	2006–2009	Multiple Regression	VAIC	ROE, ROA, GR, EP

**Fig. 1:**
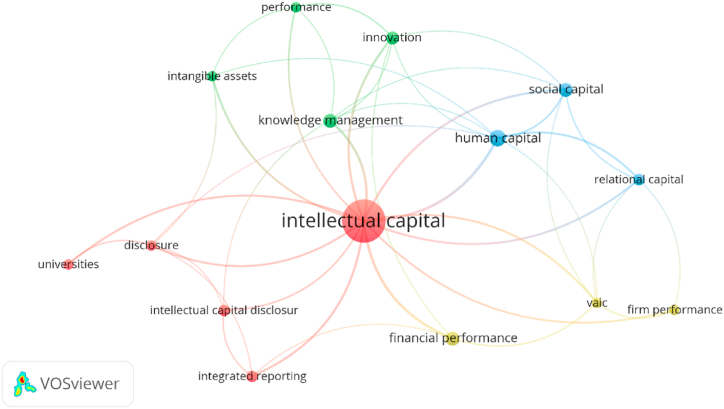
Results of the co-word analysis.

A systematic review of the previously published articles is a great avenue to understand the trends and growth in the field. For review, we have chosen the papers published in the JIC (Journal of Intellectual Capital) as the publisher is the leading outlet with a remarkable contribution to the field of study [57]. We have collected data for the period 2017–2021. Following [35], we have searched the term “ISSN (1469–1930)" in the Google Scholar database to absorb all the papers published under the JIC during the sample period. We have filtered our search to English papers only for consistency, and the papers appeared in the first ten pages for convenience. In our next stage, papers not directly relevant to our study were weeded out. Our final sample consisted of 89 articles. VOSviewer software version 1.6.18 was used for the co-wording analysis of the keywords [35]. In Fig. 1, the affinity of the keywords is visualized graphically, where stronger relations depict a shorter distance between them. Another interesting feature of the VOSviewer software is that it enables the clustering of a topic based on similarities observed in the papers. The generic return of the software presented us with 15 items under 4 clusters. Dots' sizes are varied based on normalized values of citations and importance concerning the field of study [58]. From the co-word analysis, we have found that intellectual capital is highly related to VAIC and its impact on firm performance. Hence, this study will assess the impact of VAIC on the firm's performance.

### 2.4 Central issues relating to intellectual capital and banking performance in Bangladesh

Bangladesh is on the way to becoming the South Asian knowledge hub [12] having been transformed from a labor-intensive to a knowledge-intensive economy. The banking sector leads the country as the financier of virtually all sorts of industrial activities. Yet, there are mentionable obstacles like a lack of proper governance, an increase in non-performing loans, and a crisis of capital and liquidity [59]. Additionally, social trust is low [60]. The regulating agencies regularly inject funds into the sector to tackle the issue of inadequate capital, which is not satisfactory [59] in terms of the outcomes derived. Such consequences result in poor bank performance and bank failure. Without identifying the key resources that are contributing to the bank's performance, the overall economy may fall into stagnation.

Past studies have found a marked impact of IC on enhancing the bank's performance through achieving competitive advantages [17] in Bangladesh. However, the listed banks in Bangladesh have low IC performance and a low disclosure trend in comparison to the banking industries of other emerging economies [17]. Reference [61] urges that the BSEC (Bangladesh Securities and Exchange Commission) formulate reporting guidelines that require disclosure of IC components in the annual reports of the respective banks.

In order to withstand externalities and competition, firms need intangible capabilities [62]. The central assumption of RBV theory suggests that IC shields in times of economic turmoil through intangible assets such as customer loyalty, patents [63], and the skills inherent in the human resources of the firm [64]. These assets possess unique attributes that cannot be easily imitated [65]. As a result, the firm gains competitive advantages [66]. Competitive advantage is defined as the potential to offer superior output compared to competitors within the same industry using the attributes and resources a firm owns [67]. Among the three dimensions of competitive advantages such as differentiation, outreach level, and cost leadership strategy [68], IC harnesses more on the differentiation strategy [62]. Achieving competitive advantages in effect ensures boosted firm performance [12,69].

Despite the fact that intangible capabilities are a greater source of sustainable competitive advantages [70] and require less investment than tangible assets [69], there are alternative views. Reference [71] suggests that disclosing more information about IC will position the banking industry in a better place. However, Ref. [72] finds that banking companies use their own discretion on the level of IC disclosure. In the presence of information asymmetry, investors will also fail to identify the economic downturn even if there are higher level IC disclosures [73]. Reference [63] supports this argument and criticizes the idea that higher IC firms will not be capable of enduring economic downturns.

Based on these arguments, this study offers a thorough assessment of the relationships between IC and bank performance in Bangladesh. More specifically, we are looking for the validity of the statement that intangibles can create more value for the banking companies in Bangladesh [13,74].

### 2.5 Development of testable hypothesis

Previous studies have found a significant positive impact of IC on firm performance [17,44,75,76]. We have measured performance under two aspects; profitability- ROA (Return on Asset), ROE (Return on Equity) and EP (Employee Productivity). In addition, the various components of IC were also found to be significant in the previous studies, and the sign was found to be positive; HCE (Human Capital Efficiency) [17,77,78], SCE (Structural Capital Efficiency) [53,75] and CEE (Capital Employed Efficiency) [17,53]. Details of the variables are provided in the next chapter of our study, along with sources. Reference [79] hypothesized that there exists a positive relationship between intellectual capital and firm performance. Drawing reference to the resource-based and resource dependency theories, we have also assumed the following hypotheses to test our study model:

H1 *MVAIC positively impacts t*he firm's *performance in Bangladesh with significance.*

H2 *HCE positively impacts the* firm's *performance in Bangladesh with significance.*

H3 *SCE positively affects the* firm's *performance in Bangladesh with significance.*

H4 *CEE positively influences the* firm's *performance in Bangladesh with significance.*

H5 *RCE positively influences the* firm's *performance in Bangladesh with significance.*

## 3 Research methodology

### 3.1 Data

**Fig. 2:**
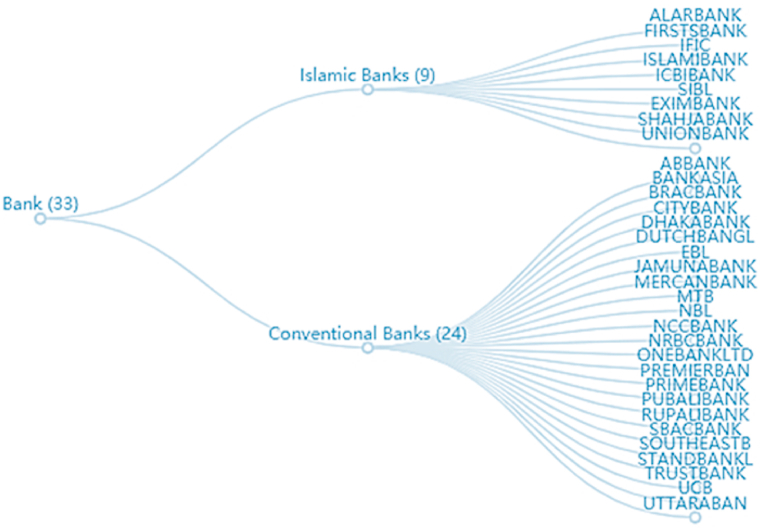
Tree graph of DSE listed Banks using Ticker symbols (Source: www.dsebd.org).

DSE is the major stock exchange in Bangladesh [80]. There are 33 listed banks on the stock exchange as can be seen in Fig. 2. We have selected all the listed banks on the DSE (Dhaka Stock Exchange) from 2015 to 2021. Our selected timeframe considers the impact of the COVID-19 pandemic on the banking industry. On July 22, 2013, the last amendment to the Banking Company Act^1^ was made, for which we chose the year 2015 as the starting year of our study. We have only considered the banking industry, as the corporate governance reports are well maintained and reported. In addition, data for the banking industry is more easily accessible through respective company websites than in any other industry in Bangladesh [81]. Therefore, we have collected data regarding IC and firm-specific control variables from the published annual reports of the firms along with the Amarstock (www.amarstock.com) and WSJ (www.wsj.com) websites. Our final sample consists of 231 bank-year observations. Throughout our dataset, we have only observed a single negative MVAIC for ICB Islamic Bank during 2015, which was due to the negative value of the SCE [82,83]. Following previous studies, we have included the negative value of MVAIC within our study [17,84,85].

### 3.2 Measurement and description of the key variables

#### 3.2.1 Dependent variables

##### 3.2.1.1 Firm performance

Firm performances can be measured in several aspects [86]. We have used two different aspects to measure performance. Profitability as the first measure of performance includes two accounting-based measures following previous studies [2,10,20,54,[87], [88], [89]]. We have also measured another aspect of performance: productivity [44,89].

##### 3.2.1.2 Measures of profitability

ROA = [Profit after tax/Total Assets] [1,53,90].

ROE = [Profit after tax/Total Shareholder's Equity] [1,53].

##### 3.2.1.3 Measure of productivity

EP = [Natural logarithm of EP], which is measured as the ratio between Profit before tax and the Total number of employees [44,88,89]. We have normalized the value of employee productivity as the number of employees significantly differs between years and banks within the industry.

#### 3.2.2 Independent variables

##### 3.2.2.1 Measuring intellectual capital

Reference [91] reviewed various models of IC measurement and pointed out that all of these are limited to a country-specific dataset. Reference [92] also reviewed the various models of IC valuation. They commented that out of numerous models, there is no universal solution to measure IC due to the presence of merits and demerits specifically attributable to specific models. Reference [88] argued that disclosure-based measures of IC are inefficient as the regulatory authorities often restrict the firms' capability to report their financial statements. Reference [93] pointed out that the perceived cost of gaining information about such intangibles from disclosures exceeds the benefit. The scarcity of disclosures published by the firms limits the scope for research on the relationship between the firm's performance and IC [88]. Alternative measures of disclosure-based IC that have been used in several studies are the VAIC model [10,44,87,[94], [95], [96]], originally developed by Pulic in 1998, which is a widely accepted and used measure for measuring the efficiency of firms in terms of their use of available resources [88]. VAIC is superior to IC disclosure-based measures as it does not suffer from any problem of judgment bias that arises during the quantification of IC disclosures and is based upon publicly accessible data [88]. However, various authors criticized the VAIC model, indicating that the measures capture the value of IC [97]. Various studies have been conducted later to solve the limitations [98,99]. Nadeem et al. (2019a) proposed a new model to overcome the previously pointed limitations through their A-VAIC (Adjusted VAIC) model. Reference [1] used both VAIC and A-VAIC models to assess the performance of the Indonesian banks and pointed out that there is still a need for a robust measure of IC components. Another popular method to measure IC is MVAIC [100,101].

We have used the MVAIC model to measure IC, which overcomes the limitations of past studies [20,90,102]. The calculation is divided into three parts [100]. In the first step, VA (Value-Added) is calculated. VA is considered an objective measurement of business achievement and indicates the ability of firms to create value. Reference [103] describes the calculation of VA as the prime concern upon which other measures are developed.

VA (Value Added) = Total Sales (OUT) – Cost of materials, services and components bought-in (IN) = Operating profit (OP) + Employment Cost (EC) + Total Depreciation and Amortization (TD&A).

In the next step, ICE (Intellectual Capital Efficiency) is measured. ICE = HCE + SCE + RCE; Where HCE is Human Capital Efficiencies, SCE is Structural Capital Efficiencies, and RCE is Relational Capital Efficiencies.

Following [103,104] investment in HC (Human Capital) should be considered an investment, not cost. HC is represented as the salaries and wages paid to the employees annually. HCE (Human Capital Efficiency) of a firm can be calculated as follows:

HCE = VA/HC;

where VA is Value Added and HC is total wages and salaries paid to employees over the year.

SC (Structural Capital) can be determined after deducting the HC from the VA. A higher share of HC in VA will result in lower SC. In addition, over expenditure in HC might exceed the Value of VA. In return, the SC will become meaningless [103] SCE (Structural Capital Efficiency) of a firm can be measured as follows:

SCE = SC/VA;

where SC is Structural Capital, and VA is the total value Added.

RC (Relational Capital) represents Marketing, selling and advertising expenses, which is used to measure RCE (Relational Capital Efficiencies). Inclusion of RCE overcomes the limitation of past studies in Bangladesh [17]. RCE represents value creation from each unit of RC, which is measured as below:

RCE = RC/VA;

where RC is Relational Capital and VA is the total Value added [100].

Finally, CEE (Capital Employed Efficiency) is measured. In addition to knowledge-based measures [103] argued that including financial and physical capital will provide proper information about the efficiency measure of value creation. CE (Capital Employed) includes the book value of a company's net assets or the shareholder's equity. Value added through the knowledge-based measure of IC is possible with the help of the CE. CEE can be measured as follows:

CEE = VA/CE;

where VA is total value Added, and CE is total capital Employed.

All the previous measures are added together for a comprehensive measure of the total value created. MVAIC (Modified-Value Added Intellectual Coefficient) enables various stakeholders to assess and compare the firm's performance with reliability and objectivity. The MVAIC can be computed as follows:

MVAIC (Modified-Value Added Intellectual Coefficient) = ICE + CEE;

where ICE is Intellectual Capital Efficiency and CEE is Capital Employed Efficiency. In summary, MVAIC will measure the value added per unit of money invested in individual resources.

##### 3.2.2.2 Control variables

BGDIV (Board Gender Diversity) = BGDIV is measured as the proportion of the number of female directors to the total number of directors on the board. Board Gender Diversity is widely used as a variable in the study of intellectual capital [55,90].

BSIZE (Board Size) = BSIZE is measured as the natural logarithm of the total number of directors on the board [55,90].

BIND (Board Independence) = BIND is measured as the total number of independent directors to the total number of directors on the board [55,90].

ASIZE (Audit Committee Size) = ASIZE is measured as the natural logarithm of the total number of members in the audit committee [105,106].

AMT (Audit Committee Meeting) = AMT is measured as the natural logarithm of the total number of audit committee meeting held in a year [106,107].

BMT (Board Meeting) = BMT is measured as the natural logarithm of the total number of the board meeting held in a year [55,108].

AGE = Firm age is measured as the natural logarithm of total firm age [90].

COVID = Value of COVID ranges from 1 if the year is 2020–2021; Otherwise, 0 [44,108]. The first case of COVID-19 was first observed on 8^th^ March 2020. An overview of the variables used in the study can be also found in Table 3.

**Table 2:** PCA analysis.

Component	Total	Percentage of variance	Cumulative percentage
Panel A: Output of the extracted component factors			
1	2.12	35.34	35.34
2	1.49	24.81	60.15

Variables	Components		Communalities
	1	2	
Panel B: Component analysis factor matrix (rotation: unrotated)			
BGDIV	−0.5522	−0.064	0.6909
BSIZE	0.7796	−0.2274	0.3406
BIND	−0.6153	0.4752	0.3956
BMT	0.3595	0.7319	0.3351
ASIZE	0.7827	−0.0884	0.3796
AMT	0.295	0.8147	0.2492
Eigenvalues	2.12	1.49	

Variables	Components		Communalities
	1	2	
Panel C: Component analysis factor matrix (rotation: VARIMAX)			
BGDIV	−0.4943	−0.2545	0.6909
BSIZE	0.8097	0.0619	0.3406
BIND	−0.7433	0.2279	0.3956
BMT	0.0786	0.8116	0.3351
ASIZE	0.7636	0.193	0.3796
AMT	−0.011	0.8664	0.2492
Eigenvalues	2.04	1.57

**Table 3:** Variable description.

Variable Name	Label	Description	Sources of Data	Sources of Variable
Dependent variable				
*Profitability*				
Return on Asset	ROA	The ratio of Profit after tax/Total assets	Annual reports	[1,53,90]
Return on Equity	ROE	The ratio of Profit after tax/Total Equity	Annual reports	[1,53]
*Productivity*				
Employee Productivity	EP	Natural logarithm of EP measured as the ratio of Profit before tax/Total number of employees	Annual reports	[44,88,89]
Independent variable				
Modified Value Added Intellectual Coefficient	MVAIC	MVAIC = ICE + CEE; Where, ICE = HCE + SCE + RCE	Author's Calculation	[100,101]
Human Capital Efficiency	HCE	HCE = VA/HC	Author's Calculation	[100,103,104]
Structural Capital Efficiency	SCE	SCE = SC/VA	Author's Calculation	[100,103,104]
Capital Employed Efficiency	CEE	CEE = VA/CE	Author's Calculation	[100,103,104]
Relational Capital Efficiency	RCE	RCE = RC/VA	Author's Calculation	[100,103,104]
Control Variables				
*Governance Structure*	STRUCTURE	Factor scores derived from a PCA (BGDIV, BSIZE, BIND, and ASIZE).	Author's Calculation	Author's idea
Board Gender Diversity	BGDIV	The proportion of the number of female directors to the total number of directors on the board.	Annual reports	[55,90]
Board Size	BSIZE	Natural logarithm of the total number of directors on the board.	Annual reports	56,91]
Board Independence	BIND	The total number of independent directors to the total number of directors on the board.	Annual reports	[55,90]
Audit Committee Size	ASIZE	Natural logarithm of the total number of members in the audit committee.	Annual reports	[105,106]
*Company Meetings*	MEETING	Factor scores derived from a PCA (AMT and BMT).	Author's calculation	Author's idea
Audit Committee Meeting	AMT	Natural logarithm of the total number of the audit committee meeting held in a year.	Annual reports	[106,107]
Board Meeting	BMT	Natural logarithm of the total number of the board meeting held in a year.	Annual reports	[55,108]
Firm Age	AGE	Natural logarithm of total firm age.	Annual reports	[90]
Corona Virus Disease	COVID	Dummy variable with a value of 1 if the year is 2020 or 2021; otherwise, 0. The first case of COVID-19 was observed on 8th March 2020.	Author's Calculation	[44,108]

##### 3.2.2.3 Principal component analysis (PCA) of corporate governance variables

This study uses various measures of corporate governance, following the previous literature, to control for any unobserved effects. Furthermore, this study also tests the role of corporate governance variables as moderators in the relationship between intellectual capital and firm performance. We have used the commonly used dimension reduction method, PCA, to reduce the large number of corporate governance variables into these components, which explains most of the variance within these variables. Following the standard PCA approach, we have only retained these factors that possess an eigenvalue that is greater than unity for further analysis [105,109]. From Table 2, Panel A, it is observable that the first two factors retain around 60% of the total variance. In Table 2, Panel B, and Panel C, we have reported the unrotated and rotated (VARIMAX rotation) component analysis factor matrix. Finally, we have named two factors on the basis of the rotated solution.

Factor 1 is named as STRUCTURE (Governance Structure) and includes BGDIV, BSIZE, BIND, and ASIZE to represent the variable governance structure. The other factor is named as MEETING (Company Meetings) which includes BMT and AMT that captures the corporate meeting dimension. We have used the Kaiser-Meyer-Olkin (KMO) measure of sampling adequacy for accurate application of the assumed method, which is found to be 0.62 and well above the minimum requirement of 0.5. Hence, we can use the factors with confidence for further analysis.

### 3.3 Empirical model

Our dataset is balanced panel data. We have conducted panel data analysis for our study. Panel data analysis is more advantageous than any other method as it controls multicollinearity and heteroscedasticity [[110], [111], [112]]. After running various panel data diagnostic analyses, the proper parameter estimation techniques were applied using robust standard errors clustered by firms that are consistent with autocorrelation and heteroscedasticity [113]. We have applied FE (Fixed Effect) and RE (Random Effect) parameter estimation techniques for assessing the impact of the independent variables on the various measures of firm performance using STATA version 17. FE and RE deal with heterogeneity and unobserved effects in the dataset more precisely than the simple Pooled Ordinary Least Squares method [44]. We have used the dataset to estimate the following empirical models:

Model [1] will examine the relationship between the aggregate measure of IC and firm performance:

$${Perf}_{it}=\alpha_{0}+\alpha_{1}{MVAIC}_{it}+\alpha_{2}{STRUCTURE}_{it}+\alpha_{3}{MEETING}_{it}+\alpha_{4}{AGE}_{it}+$$

[1] $$\alpha_{5}{COVID}_{it}+Year+\varepsilon_{it}$$

Furthermore, model [2] will look into the moderating role of corporate governance on the relationship between MVAIC and firm performance, following [101,114,115]:

[2] $${Perf}_{it}=\alpha_{0}+\alpha_{1}{MVAIC}_{it}+\alpha_{2}{STRUCTURE}_{it}+\alpha_{3}{MEETING}_{it}+\alpha_{4}{\left(MVAIC\;x\;STRUCTURE\right)}_{it}+\alpha_{5}{\left(MVAIC\;x\;MEETING\right)}_{it}+\alpha_{6}{AGE}_{it}+\alpha_{7}{COVID}_{it}+Year+\varepsilon_{it}$$

Model [4] will evaluate the relationship between the various components of MVAIC and firm performance:

$${Perf}_{it}=\alpha_{0}+\alpha_{1}{HCE}_{it}+{\alpha_{2}{SCE}_{it}+\alpha_{3}{CEE}_{it}+\alpha_{4}{RCE}_{it}+\alpha }_{5}{STRUCTURE}_{it}+\alpha_{6}{MEETING}_{it}+$$

[3] $$\alpha_{7}{AGE}_{it}+\alpha_{8}{COVID}_{it}+Year+\varepsilon_{it}$$

Finally, model [4] will assess the moderating role of corporate governance on the relationship between the various components of MVAIC and firm performance, following [101,114,115]:

[4] $${Perf}_{it}=\alpha_{0}+\alpha_{1}{HCE}_{it}+\alpha_{2}{SCE}_{it}+\alpha_{3}{CEE}_{it}+\alpha_{4}{RCE}_{it}+\alpha_{5}{\left(HCE\;x\;STRUCTURE\right)}_{it}+\alpha_{6}{\left(HCE\;x\;STRUCTURE\right)}_{it}+\alpha_{7}{\left(SCE\;x\;STRUCTURE\right)}_{it}+\alpha_{8}{\left(SCE\;x\;MEETING\right)}_{it}+\alpha_{9}{\left(CEE\;x\;STRUCTURE\right)}_{it}+\alpha_{10}{\left(CEE\;x\;MEETING\right)}_{it}+\alpha_{11}{\left(RCE\;x\;STRUCTURE\right)}_{it}+\alpha_{12}{\left(RCE\;x\;MEETING\right)}_{it}+\alpha_{13}{AGE}_{it}+\alpha_{14}{COVID}_{it}+Year+\varepsilon_{it}$$

where Perf is the various measures of firm performance (ROA, ROE, and EP); Year represents the dummies for year control; and ε is the error term of firm *i* at time *t*, respectively.

## 4 Results and discussion

### 4.1 Descriptive statistics

Table 4 displays our dataset's various descriptive statistics (Mean, Standard Deviation, Kurtosis, Minimum, Maximum, total number of observations, and Skewness). From the table, we can observe that the minimum and maximum values for MVAIC are between (−5.64) and (8.108) with a mean value of (4.193). The minimum value indicates the investment in IC exceeds the return in the form of earnings for the banks, which indicates only ICB Islamic Bank Limited in 2015 had a negative MVAIC [116]. On average, the banks listed on DSE performed well in terms of cost and return from their investments in IC. HCE contributes to value addition for firms more than any other component, with a mean value of (3.01) indicating that banks are highly invested in HCE. The other three components, SCE, CEE, and RCE, have a mean value of (0.695), (4.63), and (0.026). In terms of the dependent variables, ROA and ROE, which measure accounting-based performance for the firm, have quite different mean values of (0.007) and (0.103), respectively, indicating that equity-based returns were more observed than asset-based returns. In addition, EP has a mean value of (13.98) indicating that around 13.98% of the growth in bank value was due to the overall contribution of employees at the individual level during the study period.

**Table 4:** Descriptive statistics.

Variables	Obs.	Mean	Std. Dev.	Min	Max	Skew.	Kurt.
ROA	231	.007	.008	−.042	.021	−3.759	22.391
ROE	231	.103	.044	−.102	.263	−.49	4.819
EP	223	13.981	.627	10.349	15.279	−2.232	11.677
MVAIC	231	4.193	1.256	−5.64	8.108	−2.042	19.779
HCE	231	3.01	1.064	−1.514	6.882	−.797	8.179
SCE	231	.695	.747	−6.452	8.268	1.216	84.788
CEE	231	.463	.135	−.003	.818	−.925	5.251
RCE	231	.026	.053	−.195	.682	8.342	107.929
BGDIV	231	.115	.11	0	.429	.676	2.425
BSIZE	231	2.577	.322	1.792	3.045	−.578	2.594
BIND	231	.188	.09	0	.7	1.436	8.384
BMT	226	2.879	.382	1.386	3.97	−.475	4.408
AGE	231	3.046	.579	.693	3.892	−1.921	7.466
COVID	231	.286	.453	0	1	.949	1.9

### 4.2 Analysis of MVAIC scores ranking for the whole study period

**Fig. 3:**
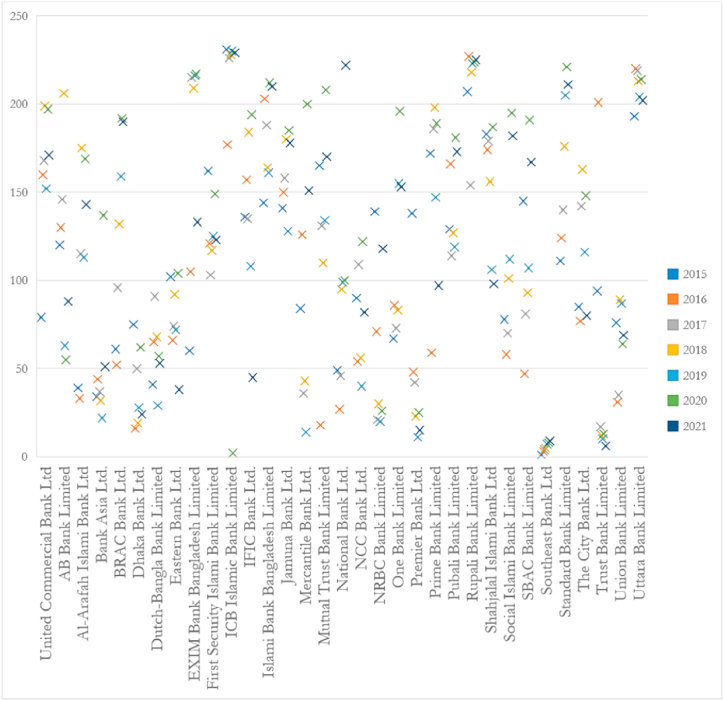
*Box and Whisker chart for MVAIC rank over the whole study period* (Source: Author).

From the dataset available, we have ranked the MVAIC scores to understand the performance trend of the listed banks over the study period in investment in intellectual capital, which can also be observed from Fig. 3. The findings suggest that Southeast Bank Limited performed well and remained constant throughout the years in terms of efficient resource utilization [117]. At the segregated level, we have found that most of the value creation for the listed banks in Bangladesh is attributable to the HCE, which is quite natural as the banking industry heavily depends upon human capital to provide customer service [118].

### 4.3 Spearman correlation analysis

We have used the Spearman correlation matrix to assess the correlation among the variables as our dataset is not normally distributed [119]. From Table 5, we find that both MVAIC and its components are significantly correlated with the dependent variables at a 1% significance level. The highest correlation with the performance measure observed is between MVAIC and EP (0.477) at an aggregate level and between HCE and ROA (0.653) at a segregated component level.

**Table 5:** Spearman correlation analysis.

Variables	ROA	ROE	EP	MVAIC	HCE	SCE	CEE	RCE	BGDIV	BSIZE	BIND	BMT	AGE	COVID
ROA	1.000													
ROE	0.617***	1.000												
EP	0.514***	0.532***	1.000											
MVAIC	0.464***	0.366***	0.477***	1.000										
HCE	0.653***	0.382***	0.467***	0.832***	1.000									
SCE	−0.248***	−0.023	0.548***	0.459***	−0.103	1.000								
CEE	0.515***	0.524***	0.062	0.387***	0.438***	−0.149**	1.000							
RCE	0.074	−0.010	−0.058	−0.462***	−0.039	−0.779***	−0.071	1.000						
BGDIV	−0.214***	−0.053	0.085	0.055	0.003	0.111*	−0.141**	0.049	1.000					
BSIZE	0.285***	0.046	0.235***	0.042	0.124*	−0.117*	0.107*	−0.127*	−0.179***	1.000				
BIND	−0.002	0.061	−0.162**	0.035	−0.015	0.055	0.104	0.107*	0.224***	−0.424***	1.000			
BMT	0.406***	0.063	0.009	0.232***	0.389***	−0.202***	0.221***	−0.003	−0.103	0.138**	0.025	1.000		
AGE	−0.291***	−0.303***	−0.255***	−0.220***	−0.261***	0.003	−0.014	0.017	−0.046	−0.206***	0.256***	0.171***	1.000	
COVID	−0.100	−0.138**	−0.086	−0.067	−0.101	0.060	−0.142**	−0.057	0.050	−0.009	0.082	−0.185***	0.153**	1.000
***p < .01, **p < .05, *p < .1														

However, we find no existence of any correlation coefficient value greater than 0.80, indicating that there is no problem of multicollinearity, excluding the cases of MVAIC and HCE. Nonetheless, the correlation coefficient value between MVAIC and HCE (0.832) is slightly over the cut-off point, which will still not be an issue as these variables were included in models separately [44]. In addition, we have also deployed the VIF (Variance Inflation Factor) to check for multicollinearity problems for further confirmation. In all cases, the value of VIF (untabulated) is within the range of the cut-off value of 10 [120,121]. Hence, from the Spearman correlation matrix and VIF test, we confirm that our dataset is free from multicollinearity.

### 4.4 Panel data analysis results

#### 4.4.1 MVAIC and firm performance

Table 6 lists the output derived from panel data estimation for our specified Eqs. [1], [2], [3], [4]. Several diagnostic tests follow our choice of the proper parameter estimation technique. The output displays that MVAIC has a significantly positive relationship with all the measures of firm performance. With reference to the RBV theory, IC efficiency is found to be driving the firm's performance. Furthermore, the EP shows the maximum coefficient (0.327) at a 1% significance level with a robust standard error (0.0848). Our result is consistent with the previous findings [13,74,122,123] which have also used fixed effect or random effect parameter estimators. In addition, we have found COVID to negatively affect ROA, with a significance that might indicate that the pandemic reduced the earning potential of employed assets. However, the effect of the corporate governance variables is not worth mentioning, indicating the ineffectiveness of the various corporate mechanisms in Bangladesh. Finally, the moderating role of two corporate governance variables constructed using PCA is not significant, which further validates the statement.

**Table 6:** Regression output (MVAIC and Firm performance).

	(1)	(2)	(3)	(4)	(5)	(6)
	ROA	ROA	ROE	ROE	EP	EP
MVAIC	.0008**	.0002	.0092*	.0175***	.3268***	.3221***
	(.0004)	(.0011)	(.0051)	(.006)	(.0848)	(.0832)
STRUCTURE	.0008	.0039	.0007	−.0039	.213***	.1406
	(.0006)	(.0033)	(.0034)	(.0307)	(.0679)	(.4589)
MEETING	.001*	.0022	.0075**	−.0147	.029	.2743
	(.0005)	(.0015)	(.0038)	(.0196)	(.0558)	(.3707)
AGE	−.0014	−.0015	−.0245**	−.0231	−.177*	−.1761*
	(.0016)	(.0018)	(.0101)	(.0142)	(.0952)	(.0949)
COVID	−.0024*	−.0023*	.0002	.002	.113	.1119
	(.0013)	(.0013)	(.0103)	(.0123)	(.14)	(.1366)
MVAIC x STRUCTURE		−.0007		.0025		.0184
		(.0007)		(.0061)		(.1093)
MVAIC x MEETING		−.0003		.0059		−.058
		(.0003)		(.0041)		(.0823)
CONSTANT	.0093**	.0124**	.1346***	.093**	12.9733***	12.9966***
	(.0039)	(.0055)	(.0412)	(.0454)	(.4654)	(.4589)
Year Dummies?	YES	YES	YES	YES	YES	YES
Observations	226	226	226	226	218	218
R^2^	0.232	0.273	0.253	0.198	0.404	0.375
Hausman Specification test						
Chi^2^-Statistic	18.62	80.04	4.17	8.22	3.96	11.23
P-value > Chi^2^	0.045	0.000	0.939	0.768	0.949	0.509

Note: This table presents the output of Panel Data regression analysis (Fixed effect and Random effect) for MVAIC and Firm Performance. Hausman test determines the choice between FE/RE estimates. Robust standard errors clustered by bank are reported in the parenthesis, consistent with autocorrelation and heteroscedasticity.

Robust standard errors are in parentheses.

***p < .01, **p < .05, *p < .1.

#### 4.4.2 MVAIC components and firm performance

In Table 7, components of MVAIC are regressed with the performance measures, which provides interesting findings. Here, we observe that HCE only holds a significantly positive relationship with ROA at a 5% significance level. The HCE does not significantly influence the other two measures of performance. Reference [44] also failed to show any evidence of a significant relationship with respect to ROA and EP. On the other hand, SCE is a significant predictor of firm performance, except ROE, which has a higher level of significance [75,124]. However, our findings contradict those of a few other authors who have failed to find any significant relationship [44,51,77,125]. CEE positively affects both ROA and ROE with significance, while in the case of EP, the relationship is not significant. We find evidence of a positive correlation, like previous studies, which confirms a relationship between the variables more clearly [75,77,125]. Our findings are opposite to Ref. [44] in terms of EP, who have found a positive correlation. RCE is only significantly and positively related to ROA at a 1% level of significance. Our study further looks into the moderating role of corporate governance variables with IC at the component level, which produces mixed outcomes. This study finds that STRUCTURE significantly and positively moderates the relationship between HCE and all aspects of firm performance, indicating that HCE enhances firm performance in the presence of a better governance structure. Following the resource dependency theory, HCE significantly contributes to the firm's performance with a better corporate governance structure. However, the moderating role is negative and significant in terms of SCE for all measures except ROE at the 5% level of significance. This suggests that structural capital efficiency is negatively affected with respect to increased board representation. Furthermore, CEE is similar to SCE, but the effect is true only for ROA and ROE. In terms of MEETING, the moderating role is significant only with ROA and the negative one except for SCE, which is not significant. The findings suggest that an increase in the number of corporate meetings negatively affects the contributions of IC, and this leads to the practicality of frequent corporate meetings. We have also observed that RCE is positively moderated in the presence of increased corporate meetings at a 1% level of significance.

**Table 7:** Regression output (MVAIC components and Firm performance).

	(1)	(2)	(3)	(4)	(5)	(6)
	ROA	ROA	ROE	ROE	EP	EP
HCE	.002**	.0016**	.0043	.002	−.1171	−.1709
	(.0008)	(.0006)	(.005)	(.0057)	(.1733)	(.1582)
SCE	.0039***	.004	.0025	.0611	4.7938***	5.0599***
	(.0014)	(.0026)	(.0056)	(.0438)	(1.7839)	(1.4086)
CEE	.0086*	.0029	.1866***	.1663***	−.0856	.0852
	(.005)	(.0032)	(.0286)	(.026)	(.4659)	(.5535)
RCE	.0679***	.0118	.0081	.1607	1.5531	2.916
	(.0226)	(.029)	(.0796)	(.1589)	(2.5827)	(2.6947)
STRUCTURE	.0006	.0059***	0	.0073	.222***	1.15*
	(.0006)	(.0015)	(.0039)	(.0153)	(.0757)	(.6071)
MEETING	.0007	.006***	.0042	−.0001	.0313	.6486*
	(.0005)	(.0012)	(.003)	(.0113)	(.0569)	(.3783)
AGE	−.0035**	−.003**	−.0244***	−.019**	−.1145	−.142
	(.0015)	(.0014)	(.0094)	(.0086)	(.0999)	(.1144)
COVID	−.0007	−.0002	−.0015	.0025	.1668	.1673
	(.0011)	(.0012)	(.0088)	(.0105)	(.1406)	(.1219)
HCE x STRUCTURE		.0008*		.0146***		.3086**
		(.0004)		(.0049)		(.1473)
SCE x STRUCTURE		−.0038**		−.0342		−3.0232**
		(.0015)		(.0336)		(1.4621)
CEE x STRUCTURE		−.0094***		−.0554***		.2778
		(.0023)		(.0204)		(.2253)
RCE x STRUCTURE		−.0478***		−.0337		.3275
		(.0161)		(.0874)		(1.4026)
HCE x MEETING		−.0008**		−.0008		.0327
		(.0003)		(.0059)		(.0995)
SCE x MEETING		.0006		.0357		−1.1507
		(.0013)		(.0298)		(.869)
CEE x MEETING		−.0052**		−.0298		−.028
		(.002)		(.0216)		(.3195)
RCE x MEETING		−.0306		−.0326		2.7688**
		(.0194)		(.1427)		(1.1798)
CONSTANT	.0039	.0071*	.0779**	.0301	11.3436***	11.3361***
	(.0049)	(.0037)	(.0314)	(.0281)	(.8517)	(.7198)
Observations	226	226	226	226	218	218
R^2^	0.495	0.605	0.427	0.508	0.496	0.487
Hausman Specification test						
Chi^2^-Statistic	148.81	47.84	15.19	18.66	3.75	11.98
P-value > Chi^2^	0.000	0.000	0.296	0.229	0.994	0.801

Note: This table presents the output of Panel Data regression analysis (Fixed effect and Random effect) for MVAIC components and Firm Performance. Hausman test determines the choice between FE/RE estimates. Robust standard errors clustered by bank are reported in the parenthesis, consistent with autocorrelation and heteroscedasticity.

Robust standard errors are in parentheses.

***p < .01, **p < .05, *p < .1.

### 4.5 Robustness check

#### 4.5.1 Dynamic model for checking the robustness of the static models

##### 4.5.1.1 Two-step robust system GMM

Following [107,126], we have also allowed for concern over endogeneity. We have further tested the dynamic nature of our previous relationship for meaningful insights using the Dynamic GMM (Generalized Method of Moments) method as suggested by Ref. [44]. Dynamic GMM addresses the presence of any simultaneity or unobserved heterogeneity. For Eqs,s. [1], [2], [3], [4], we have used static parameter estimation techniques, which may be biased as they ignore the effect of past performance, and may generate spurious results. We have used fixed effect and random effect parameter estimates in the previous section, which are only valid if the current year's performance is firmly indifferent to the past year's [126]. Reference [3] also argues that the past values of the dependent variables might also be a potential regressor for proper model estimation. For the argument mentioned above, we have also used two-step robust system GMM techniques to explore the dynamic nature of our model. We have used the [127] small sample adjustment for the downward bias of the standard errors reported in the two-step system GMM estimation.

The selection of the optimal level of lag for firm performance measures is an issue while estimating the parameters. Previous studies with small samples have ignored the test for choosing the optimal level of lag to include in their model. In contrast, we have tested for optimal lag levels following [55] and found that the impact of older lags becomes incorporated in the first lag at different levels, such as ROA (3), ROE (5), and EP (2). For which we have also used the first lag in the equation. We have estimated our model using the [128] system GMM using the xtabond2 command in STATA as prescribed by Ref. [129], with the robust option for [127] correction.

###### 4.5.1.1.1 MVAIC and firm performance

Our estimation reliability is reported at the bottom of both Table 8 and Table 9, which indicate various measures for the reliability of GMM estimation. We find that the tests for autocorrelation AR1 and AR2 remain valid as, in all cases, there is no second-order autocorrelation, which is a requirement for the GMM technique [55]. In addition, we have failed to reject the null hypotheses of the Hansen J. test, which measures instrument over-identification. Hence, all the instruments are exogenous and identified correctly. We have addressed the presence of heterogeneity that was unobserved in static parameter estimation techniques. The output of the system GMM is consistent and not biased, as it can deal with endogeneity through the use of internal instruments [129]. We find the direction and significance of MVAIC with various performance measures with the previously predicted static models remain the same with the output of the system GMM, which is a dynamic one, except with ROE. The moderating role of MEETING between the impact of MVAIC on ROA and ROE becomes positive and negative with significance, respectively.

**Table 8:** Tow-step system GMM output (MVAIC and Firm performance).

	(1)	(2)	(3)	(4)	(5)	(6)
	ROA	ROA	ROE	ROE	EP	EP
L.ROA	.335***	.366***				
	(.081)	(.084)				
L.ROE			.337***	.338***		
			(.122)	(.103)		
L.EP					.4***	.449***
					(.087)	(.095)
MVAIC	.002**	.001	.006	.009*	.203***	.164***
	(.001)	(.001)	(.006)	(.005)	(.071)	(.061)
STRUCTURE	.001	.003**	−.001	.001	.055	.124
	(.001)	(.001)	(.002)	(.013)	(.035)	(.176)
MEETING	.001	.004**	.005	−.011	−.003	.363
	(.001)	(.002)	(.003)	(.009)	(.036)	(.319)
AGE	−.002**	−.003***	−.022**	−.021***	−.09	−.033
	(.001)	(.001)	(.01)	(.008)	(.084)	(.107)
MVAIC x STRUCTURE		−.001		0		−.018
		(0)		(.003)		(.038)
MVAIC x MEETING		−.001**		.004*		−.091
		(0)		(.002)		(.076)
COVID					−.031	−.063
					(.057)	(.061)
CONSTANT	.002	.008*	.11**	.091**	7.846***	7.131***
	(.004)	(.004)	(.049)	(.038)	(1.121)	(1.406)
Year Dummies?	YES	YES	YES	YES	YES	YES
Number of Instruments	16	18	16	18	16	18
F-test (P-value)	0.000	0.000	0.000	0.000	0.000	0.000
AR (1)	0.005	0.01	0.01	0.01	0.051	0.062
AR (2)	0.627	0.311	0.105	0.095	0.398	0.458
Hansen J. test (P-value)	0.193	0.213	0.701	0.836	0.612	0.715

Robust standard errors are in parentheses.

***p < .01, **p < .05, *p < .1.

**Table 9:** Tow-step system GMM output (MVAIC component and Firm performance).

	(1)	(2)	(3)	(4)	(5)	(6)
	ROA	ROA	ROE	ROE	EP	EP
L.ROA	.464***	.295**				
	(.075)	(.142)				
L.ROE			.308***	.289***		
			(.069)	(.07)		
L.EP					.608***	.656***
					(.205)	(.147)
HCE	.002	.001	−.002	−.01***	−.128	−.161*
	(.001)	(.001)	(.004)	(.004)	(.119)	(.097)
SCE	.002**	.008	.006	.127***	2.8	3.194***
	(.001)	(.013)	(.008)	(.021)	(1.745)	(1.144)
CEE	.004	.002	.164***	.139***	.03	−.361*
	(.006)	(.003)	(.026)	(.021)	(.271)	(.203)
RCE	.019	.034***	.129	.26	.739	.981
	(.015)	(.011)	(.179)	(.229)	(1.281)	(1.216)
STRUCTURE	0.001	.01***	−.001	.038***	.055	.978*
	(0)	(.003)	(.003)	(.012)	(.046)	(.543)
MEETING	.001	.003*	.002	−.035***	.005	1.446***
	(.001)	(.002)	(.003)	(.009)	(.042)	(.272)
AGE	−.002	−.002**	−.023***	−.015**	0	.081
	(.002)	(.001)	(.008)	(.007)	(.15)	(.123)
COVID	.001	0.001	.005	.005	−.069	−.159***
	(.001)	(.001)	(.008)	(.007)	(.074)	(.059)
STRUCTURE x HCE		0.001		.013***		.169
		(.001)		(.004)		(.116)
STRUCTURE x SCE		−.01		−.076***		−1.967*
		(.009)		(.014)		(1.088)
STRUCTURE x CEE		−.008***		−.058***		−.275
		(.002)		(.019)		(.211)
STRUCTURE x RCE		−.014**		.073		−.144
		(.007)		(.093)		(.776)
MEETING x HCE		−.002*		−.009***		.033
		(.001)		(.003)		(.089)
MEETING x SCE		.007		.089***		−2.088***
		(.009)		(.014)		(.589)
MEETING x CEE		0.001		.005		−.357*
		(.002)		(.016)		(.196)
MEETING x RCE		.016		.275***		.405
		(.012)		(.084)		(1.267)
CONSTNAT	.002	.002	.06	−.006	4.036	3.148
	(.006)	(.004)	(.037)	(.029)	(2.806)	(2.339)
Year Dummies?	YES	YES	YES	YES	YES	YES
Number of Instruments	19	27	19	27	19	27
F-test (P-value)	0.000	0.000	0.000	0.000	0.000	0.000
AR (1)	0.003	0.013	0.007	0.008	0.057	0.081
AR (2)	0.459	0.451	0.143	0.233	0.334	0.271
Hansen J. test (P-value)	0.255	0.435	0.690	0.882	0.414	0.396

Robust standard errors are in parentheses.

***p < .01, **p < .05, *p < .1.

###### 4.5.1.1.2 MVAIC components and firm performance

From Table 9, we find that the output of system GMM is quite different from the static model. Although the existing significance and direction between the variables remain the same in all the cases except for HCE, the explanation power and significance level improved with the use of a dynamic estimation method. The moderating roles of STRUCTURE and MEETING do not remain the same as the static ones. In summary, the use of a static model in measuring the impact of IC on firm performance is biased and inconsistent in the case of the listed banks in Bangladesh. The past performance significantly and positively affects the current year's performance. Hence, we find that the two-step system GMM is superior in explaining the relationship.

### 4.6 Additional analysis

#### 4.6.1 Pre-Covid and during the COVID

We have used the listed bank dataset in Bangladesh for the period 2015–2021, which includes two years of COVID. We have tested the relationships before and during the pandemic to gain unique insights. From Table 10, Table 11, we can interpret that the relationship between MVAIC and firm performance was significant and positive before the pandemic and remained the same during the pandemic except for ROA. Our findings are consistent with [16]. Reference [130] found that during the pandemic outbreak, the role of IC was positive in two different countries: China and Pakistan. The authors mentioned that IC enhanced the performance of banks during the pandemic crisis. Interestingly, the relationship between the various components of MVAIC and firm performance changes before and during the pandemic, suggesting that the relationship is not dependent on the pandemic outcome. Before the pandemic outbreak, HCE and CEE significantly and positively affected ROA at a 1% level of significance. However, during the pandemic, the significance of CEE was lost. In respect to ROE, all the components of MVAIC are positive and significant, except for HCE. During the COVID outbreak, CEE only remained significant. Before the pandemic, only SCE can determine EP positively, whereas HCE and SCE are significant during the COVID outbreak. In summary, the role of IC at the aggregate and component levels differed significantly before and during the pandemic.

**Table 10:** Pre-COVID analysis.

Pre-Covid	MVAIC			Components of MVAIC		
	ROA	ROE	EP	ROA	ROE	EP
MVAIC	.003***	.011***	.287***			
	(.001)	(.003)	(.04)			
HCE				.003***	.006	.081
				(.001)	(.003)	(.106)
SCE				−.002	.029**	2.727**
				(.001)	(.012)	(1.373)
CEE				.017***	.164***	−.121
				(.005)	(.027)	(.544)
RCE				.001	.323**	1.108
				(.018)	(.133)	(1.24)
STRUCTURE	0.001	−.007**	.161**	0.001	−.002	.167**
	(0)	(.003)	(.076)	(0)	(.003)	(.071)
MEETING	.002**	0.001	−.063	.001**	−.002	−.06
	(.001)	(.004)	(.048)	(.001)	(.004)	(.048)
AGE	−.005***	−.026***	−.112	−.004***	−.025***	−.068
	(.001)	(.008)	(.085)	(.001)	(.007)	(.095)
CONSTANT	.013***	.133***	12.982***	.003	.06**	12.019***
	(.005)	(.03)	(.367)	(.004)	(.03)	(.737)
Observations	160	160	154	160	160	154
R^2^	.422	.266	.299	.62	.439	.321

Robust standard errors are in parentheses.

***p < .01, **p < .05, *p < .1.

**Table 11:** During-COVID analysis.

During COVID	MVAIC			Components of MVAIC		
	ROA	ROE	EP	ROA	ROE	EP
MVAIC	.001	.011**	.4***			
	(.001)	(.005)	(.108)			
STRUCTURE	.002**	.008	.16**	.001*	.002	.164**
	(.001)	(.005)	(.071)	(.001)	(.005)	(.069)
MEETING	.003**	.01**	.033	.002**	.007**	.02
	(.001)	(.005)	(.061)	(.001)	(.004)	(.056)
AGE	−.004**	−.014	−.223	−.002*	−.015*	−.005
	(.001)	(.011)	(.168)	(.001)	(.009)	(.136)
HCE				.002*	.001	−.419**
				(.001)	(.005)	(.171)
SCE				0.001	.007	7.583***
				(.001)	(.006)	(1.461)
CEE				.008	.211***	.225
				(.006)	(.028)	(.493)
RCE				.028	.003	1.892
				(.019)	(.15)	(1.871)
CONSTANT	.012	.089*	13.043***	.002	.046	10.256***
	(.008)	(.047)	(.644)	(.006)	(.035)	(.819)
Observations	66	66	64	66	66	64
R^2^	.403	.184	.403	.621	.527	.619

Robust standard errors are in parentheses.

***p < .01, **p < .05, *p < .1.

## 5 Conclusion

This study assessed the impact of intellectual capital efficiency on the performance of the listed banks in Bangladesh. Using the data for the DSE (Dhaka Stock Exchange) listed banks from 2015 to 2021, we have tested the impact of MVAIC along with control variables on the firm's performance. Throughout the study, we have found that the relationship between IC and performance remains almost the same in both static and dynamic models. The impact of MVAIC on firm performance is a positive and significant one, suggesting that organizations that better utilize their intangibles perform better, which is the central assumption of resource-based theory. The moderating role of meetings plays a significant role in the relationship between MVAIC and firm performance. However, at the component level, the result does not remain the same for both static and dynamic models from all perspectives. Additionally, we have found that the moderating role of corporate governance is more pronounced with respect to HCE, implying that firms that better employ governance mechanisms can significantly influence its performance. The findings are justified from the perspective of resource dependency theory, which suggests that firms with a better governance structure can access more external resources to gain competitive advantages. Further, we have tested the relationship between IC and bank performance with respect to the pre-COVID and COVID periods using static models, which remain almost the same, implying that the pandemic does not influence the contribution of IC to firm growth. This study further tests the robustness of the findings from the static models using a two-step system GMM. The explanatory power of system GMM is found to be better and more robust to interpret.

Our study has significant contributions to mention. First, we have used an alternative measure of IC rather than the disputed VAIC model, which is MVAIC, to overcome the limitations of the past studies. Second, few studies have also allowed for the proper amount of corporate governance variables in the emerging economy context. We have addressed this gap through the proper use of corporate governance-related variables as controls. Third, we have found that an insignificant amount of literature in the past has tested the moderating role of corporate governance in the relationship between IC and firm performance. Following this, we have also tested this with respect to the banking industry in Bangladesh. Finally, we address the major research gap, which is that all the papers conducted in Bangladesh used static models to determine the link between IC and performance.

Like all other studies, ours is also limited in some respects. For instance, we are limited to a single industry dataset. Future studies are expected to use multiple industries in their analysis to compare and contrast the heterogeneity present within the market simultaneously. The findings of this study have significant implications for banking sector regulation in Bangladesh. First, this study guides the regulatory authority in formulating their policy framework with respect to emphasizing the importance of IC in promoting corporate success. Second, the bank management and state policymakers can use the findings of this paper to understand the component-level impact of IC on the performance of the listed banks in Bangladesh. Moreover, the findings of this paper recommend that the Bangladesh Bank, the regulator for the banks in Bangladesh, and BSEC should work together to reshape the corporate governance guidelines, as we have found that the moderating role of governance is quite ineffective in enhancing firm performance in the presence of IC. Finally, since Bangladesh is an emerging economy in the world, the findings of this study could be generalized to other emerging economies in the world.

## Author contribution statement

Mohammad Omar Faruq: Conceived and designed the experiments; Performed the experiments; Analyzed and interpreted the data; Contributed reagents, materials, analysis tools or data; Wrote the paper.

Tamanna Akter: Performed the experiments; Analyzed and interpreted the data; Contributed reagents, materials, analysis tools or data; Wrote the paper.

Mohammed Mizanur Rahman: Performed the experiments; Analyzed and interpreted the data; Contributed reagents, materials, analysis tools or data.

## Data availability statement

Data will be made available on request.

## Declaration of competing interest

The authors declare that they have no known competing financial interests or personal relationships that could have appeared to influence the work reported in this paper.

## Footnotes

1 See Bank Company (Amendment) Act, 2013.
